# Improving Access and Confidence in Learning Lessons From Serious Incidents: A Quality Improvement Project Aimed at Junior Doctors

**DOI:** 10.1192/bjo.2022.280

**Published:** 2022-06-20

**Authors:** Cornelia Beyers, Eleanor Parkinson, Rajendra Harsh, Sameer Nardeosingh, Dolapo Oseji, Alice Packham, Nick Conway, Renarta Rowe, Ruth Scally, Joshua Rochelle-Bates, Onaiza Awais, Farhaana Surti

**Affiliations:** 1 Birmingham and Solihull Mental Health NHS Foundation Trust, Birmingham, United Kingdom; 2 Guy's and St Thomas' NHS Foundation Trust, London, United Kingdom; 3 Private, Birmingham, United Kingdom

## Abstract

**Aims:**

Birmingham and Solihull Mental Health Foundation Trust (BSMHFT) previously developed some methods of learning lessons following serious incidents. However, despite various systems available, frontline junior doctors were not regularly exposed to important learning opportunities. This potentially resulted in doctors not being aware of learning from serious incidents, and not feeling embedded within the organisation, with potential effects on their training experience. As we identified an unmet need within the Trust in learning lessons from serious incidents amongst junior doctors, we aimed to improve access and confidence in learning from serious incidents by starting a Quality Improvement project on this theme.

**Methods:**

The current approach involved a comprehensive quarterly bulletin circulated by email to staff. An initial survey confirmed that this was not very effective in delivering learning lessons information to junior doctors.

Using a QI driver diagram, we identified potential areas for change. Selected change ideas were sequentially trialled including shortened email bulletins, supervision templates and remote learning lessons sessions. Initial PDSAs highlighted difficulties with communication via email, with many trainees failing to read/engage with this format.

**Results:**

The use of remote interactive learning sessions yielded positive results, with improvement in the confidence in learning from Serious incidents. We therefore continued to refine this method to wider groups.

During the COVID-19 pandemic we experienced multiple setbacks and created a timeline tosupport team morale, maintain team energy, visualise progress and motivate the team. We therefore managed to persevere and strengthened the group by recruiting members to the team and complete the project.

**Conclusion:**

The team have been able to create a sustainable, effective and interactive short teaching session which has shown to be effective in engaging trainees in this vital area and help us meet our aim. This format further has the potential to be refined and implemented locally and nationally.

